# Complete lumbarization with calcified disc herniations at L5S1 and S1-2 levels treated with percutaneous endoscopic interlaminar discectomy: a case report and technique note

**DOI:** 10.3389/fsurg.2023.1079245

**Published:** 2023-05-18

**Authors:** Hou Lisheng, Ge Feng, Zhang Dong, Gao Tianjun, Nan Shaokui, Bai Xuedong, He Qing

**Affiliations:** ^1^ Senior Department of Orthopedics, The Fourth Medical Center of PLA General Hospital, Beijing, China; ^2^ Senior Department of Traditional Chinese Medicine, The Sixth Medical Center of PLA General Hospital, Beijing, China

**Keywords:** lumbosacral transitional vertebra (LSTV), complete lumbarization, calcified disc herniation, lumbosacral transitional disc, misdiagnosis, Castellvi classification, percutaneous endoscopic interlaminar discectomy (PEID)

## Abstract

**Objective:**

This study aims to report a case of a patient with complete lumbarization (Castellvi-IB) who developed symptomatic calcified disc herniations at L5S1 and lumbarized S1-2 levels and achieved excellent neurological recovery following percutaneous endoscopic interlaminar discectomy (PEID).

**Summary of Background Data:**

In 1984, Castellvi et al. classified lumbosacral transitional vertebra (LSTV) into four types. They incorrectly classified I LSTV anomalies as only type I sacralization, not realizing type I lumbarization also belonged to type I LSTV, with the latter exhibiting a well-developed S1-2 disc (lumbosacral transitional disc, LSTD). Patients with type I lumbarization rarely develop calcified disc herniations concomitantly at L5S1 and LSTD levels. PEID has been developed to perform discectomy for neurological decompression at the lumbar region, especially at the lowest level where the higher iliac crest and/or widened transverse process exists.

**Methods:**

A 47-year-old male presented to our hospital complaining of an intractable left leg radiating pain for 3 weeks after suffering from chronic radiating pain for 4 years. His physical examination found hyperalgesia at the lateral side of the left calf, decreased dorsal flexion strength of the ankle (grade 4/5), and a positive sign of straight leg raising test at the left side (30°). The preoperational Lumbar JOA (Japanese Orthopaedic Association) score was 12. Image examinations including whole spinal radiograph, MRI, and CT confirmed complete lumbarization (Castellvi-IB) with calcified disc herniations at L5S1 and LSTD levels at the left side. PEID was carried out at two index levels to accomplish decompression via the left approach.

**Results:**

The patient’s neurological function recovered quickly. One day postoperatively, he began to walk without discomfort. After 3 months, his muscle strength recovered to normal, and after 6 months, the residual dysesthesia at his posterolateral calf disappeared. The follow-up Lumbar JOA score was 26.

**Conclusion:**

Calcified lumbar disc herniation could develop at two distal levels concomitantly in the case of type I complete lumbarization. This anomaly might be misinterpreted as a normal lumbar sequence by only lumbar MRI. PEID may be an effective procedure to treat such calcified disc herniations in a single visit.

## Introduction

A normal spine contains seven cervical, 12 thoracic, and five lumbar vertebrae (1). Lumbosacral transitional vertebra (LSTV) is defined as either lumbarization (LZ) of the highest sacral spinal segment or sacralization of the most inferior lumbar spinal segment. LZ is a condition in which an anatomically S1 vertebra appears morphologically as a normal L5 vertebra, which creates a sixth lumbar vertebra (2). A disc just below LSTV is called a lumbosacral transitional disc (TD). It is easy to identify the existence of LSTV; however, in clinical practice, it is difficult to determine the correct segmentation or classification of LSTV just by looking at the vertebral morphology and lumbosacral axis angle shown in lumbar radiographs (2, 3). Inaccurate judgment or neglecting the existence of LSTV might occur when the segmentation is only determined from a lumbar radiograph or when a lumbar MRI is used alone (2). In 1981, Wigh et al. classified transitional discs (TD) into four types, of which a type IV TD exhibits a height and shape like a conventional L5S1 disc (4). In 1984, Castellvi et al. classified LSTV into four types, among which type I LSTV presents a large transverse process that is triangular in shape and measures at least 19 mm in width (a, unilateral; b, bilateral) (5).

TD in type I and type II LSTV belongs to type IV TD and has a well-developed disc structure similar to that of a conventional L5S1 disc in a normal spinal sequence. TD in type I LSTV (including type I LZ) (6) and type II LSTV (7) might develop into lumbar disc herniation (LDH), although very rare. In their original article, Castellvi et al. (5) and Wigh et al. (4) confused type I LZ with type I sacralization, assumed that TD in both type I LZ and type I sacralization was a true L5S1 disc, and neglected the existence of type I LZ resulting to the classification of all type I LSTV as false LSTV (“forme fruste”). While in reality, type I LZ exists as a true LSTV with an additional lumbar vertebra formation (L6) and a well-developed S1-2 disc. In addition, Casetllvi's traditional LSTV classification, there exists another special type of LZ whose transverse processes showed no difference from those at the normal L5 vertebra. In Castellvi's original article, four such cases were found and termed as full LZ, but no LDH was found at the lumbarized S1-2 disc as reported (5). There is a high possibility that full LZ was neglected or missed as a normal spinal sequence when only a lumbar radiograph was analyzed. For example, the designation L4/5 was taken as the definition of the penultimate free disc level by Ruetten et al., no matter what the correct level was (8). Lee et al. (2) provided a new classification of LSTV based on whole spinal images, in which LZ was classified into the following three types: (1) A complete LZ is when both transverse processes of the S1 do not form a joint with the transverse process of the S2 and appeared to be separated. According to this principle, both Castellvi's type I and full LZ belong to Lee's classification of a complete LZ. (2) An incomplete LZ is when both transverse processes of the S1 form a pseudoarthrosis with the transverse process of the S2 or when only one side forms a pseudoarthrosis and the other side shows a normal fusion. (3) A mixed LZ is when one side is a complete type and the other is incomplete or normal.

Although LSTD in type I LZ cases (i.e., S1-2 disc) belonged to type IV LSTD and might develop disc herniation (6), disc herniations that concomitantly developed at L5S1 and LSTD (well-developed S1-2 discs) levels were rarely reported.

Endoscopic spine surgery could provide safe, direct, and targeted access to the compressive pathology with minimal soft tissue trauma in the lumbar region (9) and, theoretically, could be accepted to deal with LDH at TD in type I LZ cases. Percutaneous endoscopic lumbar discectomy (PELD), using either the transforaminal (PETD) or interlaminar (PEID) approach, exhibits superiority over conventional disc operations, e.g., unilateral biportal endoscopic (UBE) discectomy or lumbar fusion surgery, and has been standardized as a representative minimally invasive spine surgical technique for LDH.

This study reports a case of type I LZ [Lee's complete LZ (2)] in a patient who developed calcified LDHs (CLDHs) at L5S1 and lumbarized S1-2 levels concomitantly and was treated with percutaneous PEID technique.

## Case report

**Figure 1:**
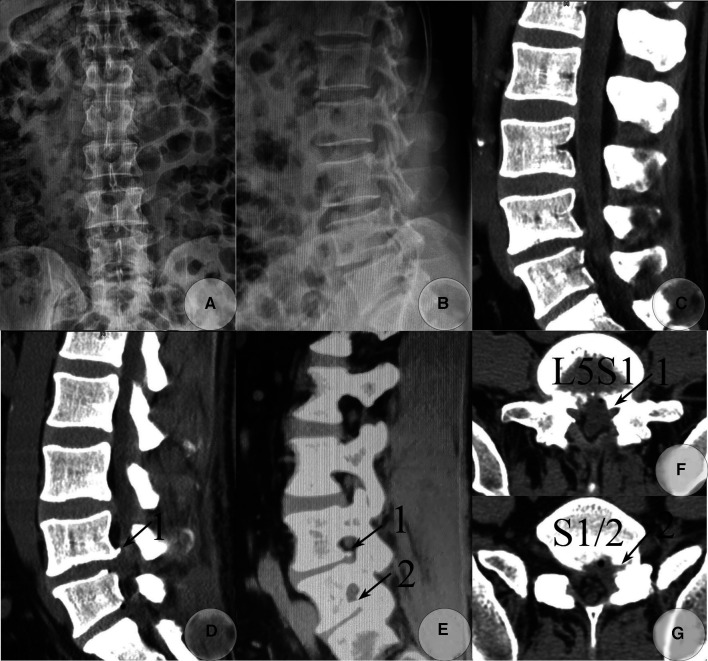
Postoperative lumbar radiograph and CT images taken on 26 and 27 December 2019, respectively. AP (**A**) and lateral view (**B**) of the lumbar radiograph. Postoperative CT images (**C–G**) detected the majority of calcified fragments that were removed. The calcified base at the posterolateral corner of the L5 inferior endplate was left asymptomatic (soft tissue window), and the calcified shell of the herniated S1-2 disc and osteophytes from the inferior endplate of S1 within the S1-2 foramen was also not removed. (**C**) Sagittal reconstructed CT image through the left paracentral plane. (**D**) Sagittal reconstructed CT image through the left recess. (**E**) Sagittal reconstructed CT image through the left foramen plane. (**F**) Raw transverse CT image through L5S1 level. (**G**) Raw transverse CT image through S1-2 level. (**Arrow 1**) Residual osteophytes at the posterolateral corner of the L5 inferior endplate. (**Arrow 2**) A residual calcified shell of the herniated S1-2 disc and osteophytes from the inferior endplate of S1 within the intervertebral foramen.

On 22 December 2019, a 47-year-old male presented to our hospital complaining of intractable left leg radiating pain for 3 weeks after suffering from intermittent chronic radiating pain close to the same region for 4 years. He had been diagnosed with LDH since the initial onset of symptoms 4 years ago as confirmed by MRI and CT examinations. No history of lumbar trauma could be recalled. Conservative treatment with lumbar traction, other physiotherapies, and some oral drugs of names he could not recall resulted in intermittent episodes of alleviation and deterioration. He had missed all medical images taken since the onset of the symptoms except the latest lumbar MRI images taken on 18 December 2019, in a local hospital, which showed left-sided LDHs at the two lowest free levels. LDH at the lowest level was a shoulder type, and that at the penultimate level mainly was an axillary type (Figure 1).

He was admitted to our department, and an intractable radiating pain from his lower back to the left buttock, the lateral thigh and calf, the dorsal and lateral plantar regions of the foot, and the great toe was noted. The back pain intensity was 8, and the leg pain intensity was 9, both measured on a 10-point visual analog scale (VAS) evaluation. His physical examination found hyperalgesia at the lateral side of the left calf, the web space between the big toe and second toe, and the dorsum of the foot, numbness at the lateral plantar aspect of the foot, decreased dorsal and plantar flexion strength of the ankle (grade 4/5), decreased dorsal flexion strength of the big toe (grade 4/5), and a positive sign of straight leg raising test at the left side (30°). The clinical signs indicated a high possibility of impingement of the normal left L5 and S1 nerves. The initial diagnosis was LDH at L4/5 and L5S1 with a normal spinal sequence (8). The preoperational Lumbar JOA (Japanese Orthopaedic Association) score was 12.

**Figure 2:**
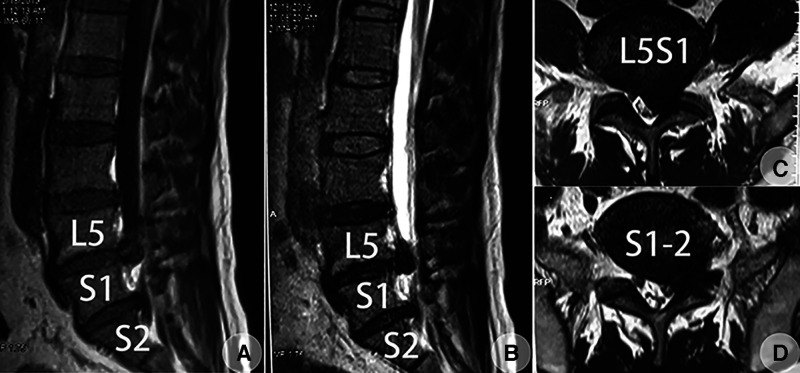
MRI of the lumbar spine taken on 22 September 2019, revealed huge LDHs at the lowest two free levels at the left side, which was later confirmed to be L5S1 and lumbarized S1-2 disc [type IV TD (lumbosacral transitional disc) according to Wigh et al.’s (4) classification based on the following whole spinal images]. (**A**) Sagittal plane through the left lateral recess (T1WI). (**B**) Sagittal plane through the left lateral recess (T2WI). (**C**) Transverse plane through the L5S1 disc (T2WI) showed an axillary type LDH. (**D**) Transverse plane through the well-developed S1-2 disc (T2WI) showed a shoulder type LDH.

Although an inaccurate judgment might occur when lumbar MRI was used alone (2), radiographs of the whole spine were taken on 23 December and revealed a type IB LZ (5) with a type IV TD (4), the so-called complete LZ as classified by Lee et al. (2). The diagnosis was corrected to LDH at L5S1 and S1-2 level with complete LZ. Both L5S1 and S1-2 levels had a wide laminar space, defined as the interlaminar window distance between the cranial and caudal laminae and between the middle line and mediodorsal border of the inferior articular process (IAP) at either level which exceeded 10 mm (Figure 2C). The intercrestal line was above the impinged two levels, and the widened transverse processes of lumbarized S1 were close to the sacral ala. No lumbar instability was found. CT scanning detected scattered calcified tissues at the posterior edge of the L5S1 disc and calcified shell at that of the herniated S1-2 disc with osteophytes formation at the inferoposterior edge of the L5 and S1 vertebrae and the superoposterior edge of the S1 vertebra. The former belonged to uncompleted while the latter belonged to complete CLDH. Both calcified disc tissues and formed osteophytes were left-sided (Figure 2).

Both disc levels were assumed to be crime levels. Although it was difficult to perform endoscopic decompression using PETD at lumbarized S1-2 level for the higher iliac crest and widened transverse process of S1 (8), a wide laminar space was found at both L5S1 and S1-2 levels. PEID was scheduled to perform the decompression procedure at the two levels under local anesthesia with conscious sedation as this provides adequate analgesia and simultaneously allows continuous feedback from the patient to avoid iatrogenic nerve damage (10, 11). Under local anesthesia, it could help to judge whether only one herniated level or all two herniated levels were responsible for the patient’s complaints without the help of a selective nerve root block preoperatively.

**Figure 3:**
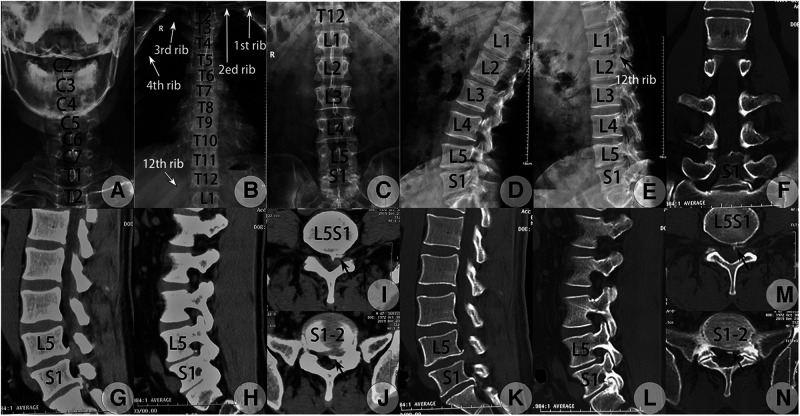
Preoperative whole spinal radiograph and CT images taken on 23 September 2019. (**A–E**) The whole spine radiograph revealed a complete LZ according to Lee's classification (2) and also type IB LZ according to Castellvi's classification (5). Anteroposterior (AP) view of the cervical (**A**), thoracic (**B**), and lumbar spine (**C**) and lateral view of the lumbar spine at flexion (**D**) and extension (**E**). The space of the interlaminar window at the sagittal or transverse direction at L5S1 and S1-2 met the criteria demand (>6 mm) for PEID (8) (**C**). (**F–N**) CT scanning revealed CLDH at L5S1 and lumbarized S1-2 levels with osteophyte formation at adjacent posterior edges, L5S1 had scattered calcified disc tissues, and S1-2 had a calcified shell at the posterior edge of herniated tissues. (**F**) Coronal reconstructed image at the bone window confirmed type IB LZ (5). Sagittal reconstructed CT image through the left lateral recess plane at the soft tissue (**G**) and bone tissue windows (**K**) detected huge soft LDH at L5S1 level with osteophyte formation at the posterior edge of the inferior endplate of L5 and a calcified shell of the herniated S1-2 disc connected with osteophytes from the posterior edge of the inferior endplate of S1 while still separated from the superior endplate of S2. Sagittal reconstructed CT image lateral to the medial border of the left pedicle at the soft tissue (**H**) and bone windows (**L**) detected scattered calcified disc tissues at L5S1 level and a calcified shell of the herniated S1-2 disc connected with osteophytes from the posterior edge of S1. Raw transverse CT image adjacent to the inferior endplate of L5 at the soft tissue (**I**) and bone windows (**M**) detected calcified tissues from the herniated L5S1 disc at the lateral region, which was separated from the L5 vertebra (black concave arrow). Raw transverse CT image at the superior endplate level of the S2 vertebra at the soft tissue (**J**) and bone windows (**N**) detected a calcified shell of the herniated S1-2 disc separated from the S2 vertebra at the left lateral margin (black concave arrow) while no osteophyte was found at the non-herniated right side.

The decompression was performed on 25 December 2019. The patient was placed in the prone position, and the L5S1 and S1-2 levels were confirmed under C-arm guidance (Figure 3A). The skin entrance points were marked (with reference to the medial pedicular line on AP and spino-laminar line on lateral view) (Figure 3B). L5S1 level was chosen as the first decompression target, as the herniated tissues at this level were mainly soft and might be the major source of the patient’s complaints (Figure 2). All the operating instruments and optics were supplied by Dragon Crown Medical Co., Ltd. (Jinan City, Shandong Province, China). While draping the operation field, the surgeon started a continuous intravenous administration of dexmedetomidine hydrochloride solution in a dose of 100 μg/ as conscious sedation (10). The level of anesthetic was titrated so that the patient could communicate with the surgeon throughout the procedure. The “V” point (the intersection of the ligamentum flavum, IAP, and superior articular process), which was approximately 1 cm lateral to the midline of the spinous process, was chosen as the docking point. Using an 18 G spinal needle, 15–20 mL of 0.5% lidocaine was injected layer-by-layer into the skin, subcutaneous tissue, fasciae, and muscle, till the needle encountered hard resistance from bony structures which were a little lateral to the V point, after optimal positioning of the needle tip under the C-arm guidance. In addition, 5–10 mL of 0.5% lidocaine was infiltrated around the periosteum and the ligamentum flavum (LF) to prevent pain from following LF splitting (Figure 3C). A longitudinal incision about 7 mm in length taking the needle as the center was made, and the subdermal fascia was dissected. A dilator, 6 mm in outer diameter, was inserted to reach the target “V” point and confirmed by hand feeling. A beveled-ended tip working channel, 6.3 mm in inner diameter, was inserted through the dilator using the counter side beveled to avoid the bony obstacle initially, and then a rotational insertion of the working channel toward the V point was performed after contact with the LF. After optimal positioning of the working channel under C-arm guidance (Figure 3D), a 30° endoscope was inserted. The irrigation system was then connected. The cannula was rotated to push the peripheral muscles aside which eased the LF exposure. The endoscopic LF exposure was performed under continuous pressure irrigation using antibiotic-instilled normal saline (6 mL of gentamycin sulfate injection for antibiotic and 0.3 mg of adrenaline hydrochloride injection for bleeding control were added per 3,000 mL of normal saline). The irrigation pressure was 20–40 mm Hg with a 100% flow rate and was adjusted depending on the clarity of the visual field. A bipolar radiofrequency (RF) device (manufactured by Beijing Jeswis Tech. Co. Ltd., Beijing, China) was used for coagulation of the bleeding. The bony corner formed by the superior process turning to the superior edge of the inferior lamina from the S1 vertebra was identified endoscopically. The most inferolateral LF connected to this corner was exposed and split longitudinally using a probe and resected in the same direction using a punch to open a 5-mm-long window to expose the epidural space, which is located at the axilla area of the traversing S1 nerve (Figure 3E). Following the exposure of the epidural space, the irrigation was stopped, and 5–8 mL of 0.5% lidocaine was injected into the epidural space using an 18G needle via the endoscopic channel. We waited approximately 5 min for further traversing nerve and dural sac pain control. The first endoscopic sight at epidural space was herniated L5S1 disc at the axillary region of the S1 nerve as the S1 nerve had been displaced to the bony lateral recess region. After removal of the adhesion bands using RF, the cottony tissues of non-calcified or incomplete CLDH were bitten piece by piece. Then, the beveled opening of the cannula was rotated toward the midline of the spinal canal slightly to expose the herniated tissues beneath the dura sac, which were removed using a curved forceps. The small inferior calcified base at the corner of posterosuperior margin of S1's superior endplate was removed by rotating and migrating the beveled-ended tip of the cannula as an end-cut trephine. The superior calcified base at the corner of posteroinferior margin of L5's inferior endplate was left intact, because it was located ventral to the S1 nerve bifurcation and produced no compression. The working cannula was then moved to the shoulder space of the S1 nerve after soft retraction of the S1 nerve medially to remove the residual herniated disc shoulder ventral to the S1 nerve (Figure 3E–I). Medial and lateral as well as cranial and caudal mobility of the working canal within the spinal canal was controlled using optics on the joystick principle. No power burr was used due to fear of iatrogenic damage to the adjacent neural elements as we were not familiar with power burr use.

**Figure 4:**
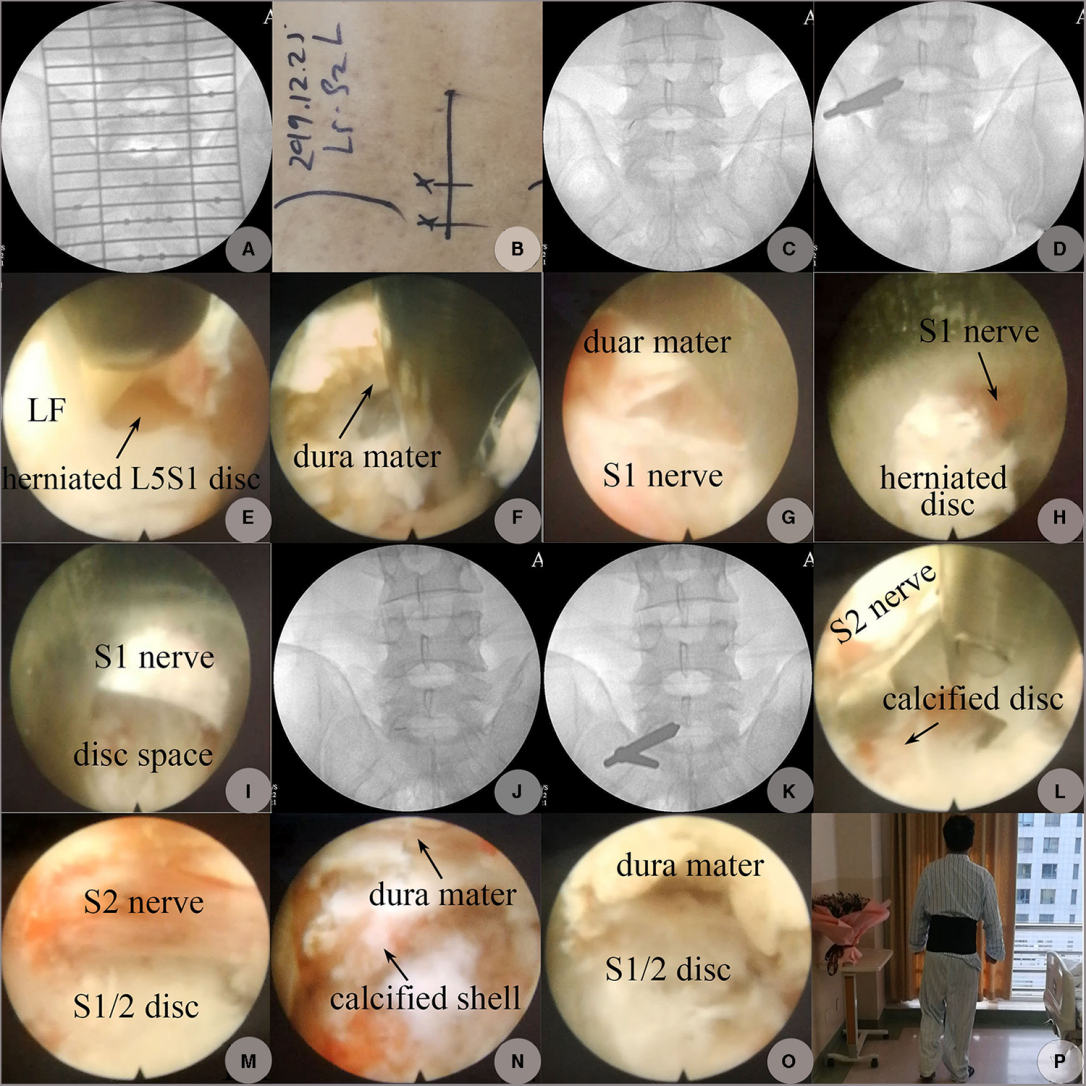
PEID performed on 25 December 2019. (**A**) AP view on C-arm fluoroscopy to determine the skin incision positions. (**B**) Skin marks for entrance points. (**C**) The location of the puncture needle tip at L5S1 level confirmed on AP fluoroscopy (“V” point: the intersection of the ligamentum flavum, IAP, and SAP). (**D**) The location of the working cannula's beveled end adjusted to the inferolateral corner formed by the medial edge of SAP and the superior edge of the lamina from S1. (**E**) LF at the latero-inferior corner of the L5S1 interlaminar space was split longitudinally exposing fragments of the herniated L5S1 disc at the axillary region. (**F**) Hard and soft fragments of the herniated disc axillary to the S1 nerve were resected using angled forceps, the dura mater was at the 12 o'clock direction, and the S1 was at the 6 o'clock direction beyond endoscopic visualization. (**G**) Free space after herniated disc fragments at the axillary region of the S1 nerve was resected, and the S1 nerve appeared at the 6 o'clock direction. (**H**) Herniated disc fragments shoulder to the S1 nerve. (**I**) Free space at the shoulder space shoulder to S1 after the herniated disc at this region was removed. (**J**) The location of the puncture needle tip at S1-2 level. (**K**) The location of the working cannula's beveled end pointed to the superolateral corner formed by the medial edge of IAP and the inferior edge of the lamina from S1. (**L**) Calcified fragments of LDH shoulder to the S2 nerve were removed using an angled forceps. (**M**) Calcified fragments of the herniated S1-2 disc shoulder to the S2 nerve were shaved off by rotating and migrating the beveled end of the working cannula as an end-bit trephine exposing free space shoulder to the traversing S2 nerve. (**N**) Residual calcified shell and submerged soft tissues of the herniated S1-2 disc axillary to the S2 nerve. (**O**) Free space beneath the dura mac at S1-2 level after decompression; the S2 nerve was at the 6 o'clock direction but beyond the endoscopic visualization. (**P**) One day postoperatively, on 26 December 2019, the patient began to walk without any discomfort; 12 o’clock was the medial direction, and 9 o’clock was the cranial direction.

The radiating pain at the anterolateral region of the left calf and dorsal aspect of the foot disappeared following complete decompression of the S1 nerve, while that at the posterolateral region of the left calf and plantar aspect of the foot was still left, which implies that the compressed S2 nerve was responsible for residual symptoms and still needed decompression. CLDH at the lumbarized S1-2 level was immediately chosen as the second decompression target. The establishment of the trajectory path was repeated at S1-2 disc level. The shoulder space to the S2 nerve was exposed first as the S2 nerve had been displaced medially by the herniated S1-2 disc which resulted in a narrowed axillary region and widened shoulder region (Figure 1D). After insertion of the working cannula, the tip of the working cannula was adjusted toward the bony corner formed by the inferior edge of the superior lamina turning to the base of the inferior process from the S1 vertebra under C-arm guidance (Figures 3J,K). The most superolateral LF connected to the superolateral bony corner of the S1-2 interlaminar space was exposed and split longitudinally and resected in the same direction to expose the shoulder area of the S2 nerve endoscopically. The S2 nerve was retracted medially using the beveled-ended tip of the working cannula as a retractor. The calcified shell of the herniated S1-2 disc was grounded off by rotating and migrating the beveled-ended tip of the working cannula. The residual non-calcified disc tissues were removed with forceps. The working cannula was then moved to the axillary region of the S2 nerve. The residual disc tissues at the axillary region were removed when pushing the S2 nerve laterally (Figures 4l–O). It was found intraoperatively that the inferior edge of the calcified herniated S1-2 disc shell had no connection with the superior endplate from S2. The radiating pain in the left calf disappeared following the full decompression and free movement of the S2 nerve. The calcified shell of CLDH and osteophytes within the S1-2 foramen, which are all located at the inferior portion of the foramen, were removed, produced no compression to neural elements nearby, and were left undisturbed (Figure 4G). No obvious bleeding was seen. The patient's residual pain disappeared. After confirming the free course of the S2 nerve root and thecal sac, the endoscope and the working cannula were withdrawn, and the opening created in the LF was seen to close spontaneously. Hence, following the further withdrawal of the cannula, the working path that was created through the muscle fibers by the dilator was visualized endoscopically to close spontaneously without creating any dead space. No drainage tube was given when ending the decompression procedure. Each skin incision was closed using a single non-absorbable suture.

After the operation, the same intravenous drugs were repeated and continued for 3 days, and 500 mL of 5% glucose injection with 2 μ of hemocoagulase agkistrodon was given by intravenous drip once per day and continued for 2 days.

The patient had a satisfactory alleviation of radiating leg pain. He had a good sleep on the operation night. One day postoperatively, the VAS scores of back pain and leg pain dropped to 1 and 0, respectively. His dorsal flexion strength of the left big toe recovered to grade 5. He began to walk without any discomfort. Straight leg raise exercises and suitable ambulation were advocated. CT re-examination on 27 December 2019, detected remnants of osteophytes at the posterolateral corner of the L5 inferior endplate with complete removal of the calcified and non-calcified tissues at other locations at L5S1 level. In addition, the calcified shell of the herniated S1-2 disc and osteophytes were removed completely from the inferior endplate of S1 within the spinal canal, while those at the inferior portion of S1-2 foramen were left but produced no compression on neural elements (Figure 4). He was discharged on 27 December 2019, with a back pain VAS score of 0. The postoperational Lumbar JOA score was 20. A lumbar support belt was used for 2 weeks. At 3 months of follow-up, his muscle strength recovered to normal. At 6 months, residual dysesthesia at the posterolateral calf disappeared. The last follow-up taken on 6 January 2023, via telephone revealed that he remained to be in a healthy state, although he felt a 3 cm×3 cm numbness region at his posterolateral calf and the lateral side of his big toe, without any other discomfort. His back pain and leg pain VAS scores remained 0. The follow-up Lumbar JOA score was 26. He was satisfied with the outcomes of the surgery, and we failed to persuade him to take a CT or MRI re-examination.

## Discussion

LSTV are common congenital anomalies (1, 2). However, only a whole spinal image could detect a complete LZ and allow differentiation between type I LZ and type I sacralization. If only a lumbar MRI was performed, it was impossible to determine the correct segmentation in 6.2% of the LSTV group (1). Full LZ might be misdiagnosed as normal spinal sequence, and confusion between type I LZ and type SZ might occur, which might result in confusion between true (type I LZ) and “forme fruste” LSTV (type I sacralization). This was confirmed by our case. Whole spinal images help detect the existence of type I LZ without incorrect level numbering. When only lumbar images were present, segments might be incorrectly considered in as high as 11% of cases (1). Some defined the penultimate free disc level as designation L4/5, which might be real L5S1 in complete lumbarization cases (4) or real L3/4 in complete sacralization cases (1).

The identiﬁcation of the most offending lumbar level is localized through a review of medical history and dermatomal distribution of pain (3). Patients with multilevel LDHs also have signiﬁcant beneﬁt from single-level PELD based on a good response to a selective nerve root block as a preoperative adjunct (7). To verify whether our case could benefit from single-level PEID, we first performed PEID at L5S1 level under local anesthesia, instead of a selective nerve block, and found that PELD at L5S1 only produced significant results at some parts of the impinged areas. In addition, PELD at S1-2 level was performed immediately with excellent outcomes. If the PELD was performed under general anesthesia, it was difficult to define whether one PELD at one level had produced satisfactory results. Reports on PEID successfully performed under local anesthesia encouraged us to perform PEID for our case under local anesthesia (3).

Patients with LSTV may have dermatome variations. Chang et al.'s study found that the S1 nerve root in LZ cases had the usual function of the L5 nerve root. The most plausible explanation of this finding was that the lumbosacral plexus was postfixed in these patients (12). The S1 nerve root contributed to the bigger cranial portion of the lumbosacral plexus (12). Our case found that the S1 nerve might have the function of the L5 nerve and the S2 nerve root might have the usual function of the S1 (L6) nerve root in a normal configuration. This is supported by the fact that when the S1 nerve was decompressed at L5S1 level, the radiating pain at the anterolateral region of the calf and dorsal aspect of the foot at the impinged side disappeared, and when the compressed S2 nerve at S1-2 level was decompressed, the radiating pain at the posterolateral region of the calf and plantar aspect of the foot disappeared. Therefore, it was difficult to find type I LZ through lumbar spinal images and neurological examination.

Chang et al. found that the disc just above LSTD had a higher tendency to develop LDH compared with that at LSTD (12). Type IV LSTD in type I LZ cases might develop huge LDH, but this was not common ^[^**^错误!未定义书签。^**^]^. Our case found a concomitant occurrence of LDH at two distal levels in type IB LZ cases. This was a rare phenomenon. Calcified disc herniation at two distal levels in type IB cases might be extremely rare.

PEID is mainly introduced to deal with LDH at L5S1 level in normal configuration, especially with high crest and/or large transverse process of the L5 vertebra, although LDH at L2-3 to L4-5 levels were sometimes chosen as candidates (8). CLDH is a special type of LDH. Specific reasons for the formation of calcification remain unclear yet (3). Patients with calcified LDH usually have a long course of conservative treatment prior to consideration of surgery. Jasper et al. considered that mineral deposit formation and even calcification might occur in the herniated nucleus pulposus when the disease course of LDH exceeded 6 months (7). Chronic inﬂammation is a possible cause (3). Dabo et al. pointed out that long-term administration of traditional Chinese medicines (TCM) might be related to CLDH (4). We had no knowledge about the exact reasons of CLDH formation in our case, but the only clear factor was the 4-year-long chronic lumbar pain disease course. The majority of LDH at L5S1 level was soft, while the majority at S1-2 level was hard. These persuaded us to accept that the LDH at L5S1 level developed at a later period.

In Chen et al.'s literature, PETD is indicated when the CLDH is at the shoulder or ventral side of the traversing nerve, and PEID is indicated when the CLDH is at the axilla of the traversing nerve (3). The CLDH at L5S1 level in our case belonged to a shoulder type and could be handled with PETD, and that at lumbarized S1-2 level belonged to an axillary type. Meanwhile, for the high iliac crest and widened transverse process, PETD was the only choice. To accommodate the CLDH at S1-2 level, PEID was chosen for the two levels. It was said that in the presence of LSTV, normal vertebral biomechanics are impaired due to the deterioration of the anatomy. Consequently, instability and early disc degeneration are observed (1). Our patient belonged to type I LZ with type IV LSTD, and no instability was observed.

According to Ruetten's description (8), if PEID was chosen, the interlaminar window between the cranial and caudal lamina and between the middle line and mediodorsal border of IAP had to be at least 6 mm. Opening of the ligament to insert the endoscope into the spinal canal can be limited to 5 mm in length. The related values at L5S1 and lumbarized S1-2 levels in our case met the indications. The interlaminar space is usually large enough for the working cannula and the endoscopy to pass through. It was also applicable to other levels by using endoscopic punches or drills to enlarge the interlaminar space (13, 14). PEID showed advantages in dealing with calcified CLDH (14). Our case was a calcified CLDH at both L5S1 and lumbarized S1-2 levels. Both the S1-2 and L5S1 interlaminar spaces were large enough for the working cannula to be inserted to either the shoulder or axillary region without difficulty (Figure 3). The use of the bevel-ended tip of the working cannula as a trephine successfully removed most calcified tissues. The goal of the treatment of calcified CLDH was not to achieve complete radiographic resection but to decompress the nerve root and dura sac effectively, while the intervertebral structure should not be disturbed excessively (14). Some calcified base linked to the posterior margin of L5's inferior endplate was left intact because it was asymptomatic. At 3–4 years of follow-up, no new osteophytes or CLDH formation was found, and good neurological recovery was maintained.

We did not use a power drill to perform the decompression due to our lack of experience in its manipulation when performing PEID while realizing the existence of many neural elements surrounding the CLDH. We only used a power drill to thin the calcified disc shell when performing endoscopic decompression via a transforaminal approach using an inside-out technique. We were not confident whether the remaining calcified intervertebral discs in L5 in our case would proceed further, but we preferred that the possibility was low. Even though the remaining CLDH proceeded further, it was an asymptomatic development as no new complaints came from the patient during the final follow-up.

UBE discectomy is a rapidly growing surgical method that uses an arthroscopic system for the treatment of LDH. It was reported that the application of UBE for the treatment of LDH yielded similar clinical outcomes to PEID. However, UBE was associated with various disadvantages relative to PELD, including increased intraoperative and hidden blood loss, longer hospital stays, more total hospitalization costs, should be performed under general anesthesia, and needing more skin incisions and extra-spinal exposure. It can also produce more soft tissue trauma and scar formation of the epidural space compared with PELD (9). In addition, UBE might lead to excessive FL removal, which was not the best choice for our case. There was no instability at the index levels. Therefore, we do not think it was suitable to perform lumbar fusion. Increased patient demand for more minimally invasive procedures can also lead to increased popularity of PELD and decreased tendency of conventional fusion (7).

We do not have enough evidence to judge whether osteophytes or disc herniation with calcification emerged first, as the patient’s medical images taken previously had been lost. However, we tended to accept the opinion that CLDH emerged first, realizing the fact that osteophytes were only found at the herniated side and no osteophytes were found at the non-herniated location in involved levels and at other non-herniated levels. If the osteophytes were the primary factor, osteophytes should have been found at non-herniated locations. Compared with soft LDH, it might need more time to develop a calcified LDH. We thought that disc herniation at L5S1 level might develop later, realizing the main portions of the herniated L5S1 disc were soft type and osteophytes were smaller compared with those at S1-2 level. This also was the reason why we chose L5S1 level as the first decompression target, realizing the calcified S1-2 disc might not be the major reason counting for the patient's complaints. If the patient's symptoms alleviated obviously at all impinged regions following the decompression at L5S1, we might abandon further decompression at S1-2 level.

Under most circumstances, PEID was performed under general anesthesia for intractable pain during retraction of the traversing nerve if only local anesthesia was given (4). While general anesthesia was introduced, it was difficult to distinguish whether the LDH at L5S1, which was mainly composed of soft herniated tissues, was the only or primary factor for the patient's symptoms or whether calcified LDH at S1-2 was also responsible.

Our case revealed that calcified LDH could develop concomitantly at two distal levels in type I LZ cases with osteophyte formation. This anomaly might be misinterpreted as a normal lumbar sequence by only lumbar MRI. PEID may be an effective procedure to treat such calcified disc herniations.

## Data Availability

The raw data supporting the conclusions of this article will be made available by the authors, without undue reservation.
